# Osteosarcomagenesis: Modeling Cancer Initiation in the Mouse

**DOI:** 10.1155/2011/694136

**Published:** 2011-02-20

**Authors:** Kevin B. Jones

**Affiliations:** Department of Orthopaedic Surgery and Center for Children's Cancer Research, Huntsman Cancer Institute, The University of Utah, Salt Lake City, UT 84112, USA

## Abstract

Osteosarcoma remains a deadly malignancy afflicting adolescents and young adults. The lack of a precursor and the panoply of genetic aberrations present in identified osteosarcomas makes study of its initiation difficult. A number of candidate hypotheses have been tested in the mouse, a species with a higher background incidence of osteosarcoma. Chemical carcinogens, external beam radiation, and bone-seeking heavy metal radioisotopes have all proven to be osteosarcomagenic in wild-type mice. A number of oncogenes, introduced via integrating viruses or aberrantly activated from heritable genetic loci, participate in and can individually drive osteosarcomagenesis. Germline and conditional gene ablations in the form of some but not all aneuploidy-inducing genes, conventional tumor suppressors, and factors that function normally in mesenchymal differentiation have also proven osteosarcomagenic, especially in combinations that silence the *Rb1* and *p53* pathways. This paper reviews the rich history of mouse models of osteosarcomagenesis, what they have taught us about the human disease, and what future mouse experiments yet promise to teach.

## 1. Introduction

Osteosarcoma is the most common primary bone malignancy and a leading cause of cancer death in adolescents and young adults [1]. Phenotypically, osteosarcoma adheres to a narrowly defined pattern of disease. 

Most osteosarcomas arise in the 2nd and 3rd decades of life in the metaphyses of long bones, especially near the major growth centers of the distal femur, proximal tibia, and proximal humerus [1, 2]. When osteosarcoma rarely develops in a patient over 40, it is usually secondary to prior radiation exposure or Paget's metabolic disease of bone. The vast majority of osteosarcomas (~95 percent) present as high-grade neoplasms, with microscopic metastatic disease at presentation the expectation in every case [3]. Intermediate and low-grade variants of osteosarcoma are extremely scarce [4]; benign bone-forming neoplasms are also much more rare than conventional osteosarcoma itself. There is no identifiable precursor to osteosarcoma.

Despite this narrow clinical phenotype, the genotype of osteosarcoma aligns best with high-grade carcinomas, by its many cytogenetic aberrations and multiple mutations. It is difficult to discern which of these many derangements are causative of, as opposed to resultant from oncogenic transformation. Naturally, when the final state of these cells fails to readily highlight the pathway of transformation that engendered them, and no precursor lesion is known, scientists turn to model systems to investigate cancer initiation hypotheses.

Mouse models of human diseases have proven useful in both mechanistic biological understanding of pathogenesis and preclinical evaluation of medical interventions. For the field of cancer research, most mouse models have been xenografts of human cells into immunocompromised mice. Recent efforts have extended to genetic mouse models of disease, given the ability to manipulate the murine genome with predictable facility. The modeling of osteosarcoma specifically in mice predates the wide availability of xenografts and even the technological innovations that permitted gene targeting in the mouse.

Although much remains unknown regarding osteosarcomagenesis, rodent models of osteosarcoma initiation have taught us much and promise to have much more yet to teach.

## 2. Random Mutagenesis and Induced Chromosomal Instability

The background rate of osteosarcomagenesis in rodents is higher than the background rate of most carcinomas and much higher than the natural incidence of osteosarcoma in humans, but is still low. Control mice in most induction studies have under five percent natural lifetime incidence of osteosarcoma. A mouse strain with a higher basal incidence of osteosarcoma was reported in 1938 but may have carried germline mutations in some important tumor suppressors yet unrecognized [5].

### 2.1. Chemical Carcinogens

The first report of chemically induced murine osteosarcomagenesis was also in 1938. Drs. Brunschwig and Bissell surgically placed crystals of 1,2-benzpyrene mixed in cholesterol into the tibial medullary cavities of a few mice. One of these formed a radiographically and histologically verified osteosarcoma 8.5 months later [6]. The same authors had previously produced nonosteogenic sarcomas by the application of 3-methylcholanthrene [7]. Many reports of chemical carcinogen-induced osteosarcomas in mice and rats have followed, but a theme that began with these first two attempts has persisted: bulky-adduct-forming heterocyclic compounds have generally proven more efficient in the induction of osteosarcomagenesis than methylating agents, which tend to produce fibrosarcomas [8–10]. 

The mechanisms by which these different classes of agents generate genetic instability are chromosomal instability from bulky-adduct-forming agents and microsatellite instability derived from a mismatch repair defect in methylating agents [11]. This observation says nothing definitive about human osteosarcomagenesis but suggests that chromosomal instability is an important element of the genetic distress and instability involved in pathogenesis.

**Figure 1:**
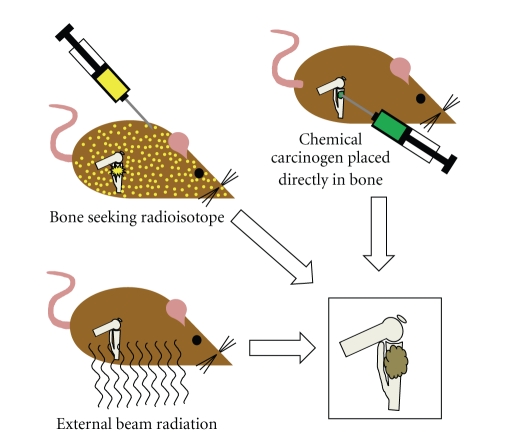
Osteosarcomagenesis can be induced in genetically wild-type mice through a variety of means. Most heavy metal radioisotopes will naturally home to ossifying bone matrix in the metabolically active metaphyses of long bones, emitting their radiation locally after embedding. External beam radiation in varied forms has also proven successfully osteosarcomagenic. Although a few chemical carcinogens administered systemically by oral or intravenous application in mice have proven sufficient, most osteosarcomagenic chemical compounds have been orthotopically implanted in the tibia or femur.

Most chemically induced mouse osteosarcomas have arisen from orthotopically implanted chemical carcinogens (Figure 1), arguing that environmental exposures to chemical carcinogens may not play a large role in sporadic human osteosarcoma incidence. One exception to this is the oral administration of 1-(2-hydroxy-ethyl)-1-nitrosurea to rats, which generated osteogenic or chondrogenic sarcomas in 58 percent. Overall, chemical implant-related osteosarcomagenesis has taught us primarily about the effects of varied methods of DNA damage, rather than necessarily recapitulating the actual etiology of the human disease. Although two reports have considered an increased incidence of osteosarcoma in pediatric and adult populations exposed to pesticides, the links are yet to be verified in second populations [12, 13].

### 2.2. Radiation

 Since the observation of frequent osteosarcomas arising among radium dial painters in 1931 [14], there have been many attempts to induce osteosarcomas by application of radiation to the rodent skeleton. Some of this work in rabbits actually predated Brunschwig's chemical induction of a mouse osteosarcoma [15, 16]. Both external beam and internal exposure to filtered and unfiltered rays from radium, thorium, and roentgen radiation have proven sufficient to induce osteosarcomas in rodents at high enough doses [17–20]. Beyond fitting the theme of DNA damage readily inducing osteosarcoma, this has an obvious correlate with human osteosarcomagenesis, as exposure to environmental or therapeutically/diagnostically applied external beam radiation is known to increase risk for osteosarcoma [21]. Osteosarcoma is one of the more common radiation-induced secondary cancers in humans.

Mice have otherwise been induced to initiate osteosarcomagenesis with radioactive heavy metals [22–30]. These ions tend naturally to home to the bone and incorporate in the hydroxy-apatite crystals that mineralize the ossifying matrix, each ion usually replacing a calcium ion in the structure. Most of the successfully osteosarcomagenic radioisotopes have been alpha emitters. One can certainly speculate as to the particular variety of genomic damage caused by alpha particles, but this observation may have had as much to do with simple anatomical localization of the damage to osteoprogenitors near the matrix mineralization front, as alpha particles do not traverse multiple tissue planes efficiently, due to their large size. Certainly, beta-emitters that home to bone have also proven osteosarcomagenic.

### 2.3. Viral Insertional Mutagenesis

Genomic distress can also be generated by infection with integrating retroviruses. Many of the early mouse models of leukemia and lymphoma were driven by the mutations caused by integrating viral DNA [31]. Cloning of these sites was the first major source of information regarding tumor suppressors and oncogenes in general. There are no reports of osteosarcomas arising in mice exposed to such insertional mutagenesis specifically, but the contribution of such insertional mutations to the osteosarcomagenesis induced by oncogene-expressing integrating viruses has not fully been explored.

### 2.4. Gene Targeting

**Figure 2:**
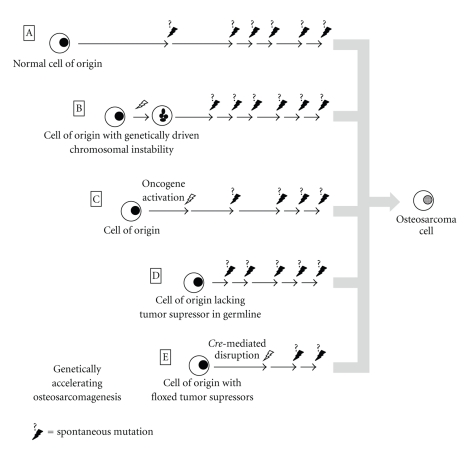
Genetically wild-type cells can naturally accrue sufficient mutations to initiate osteosarcomagenesis in mice as evidenced by the background incidence of osteosarcomas a little under 5 percent in most strains (A). Genetically induced aneuploidy alone, in most of its forms, is inefficient in osteosarcomagenesis (B). Expression of oncogenes as transgenes using native promoters, introduced via insertional viral vectors or unmasked by radiation, can lead to benign and malignant bone neoplasia in mice (C). Inherited germline deletion of tumor suppressors in heterozygosity or homozygosity or generation of mouse chimeras of cells with and without such deletions to avoid serious developmental defects have proven the efficiency of many gene inactivations in osteosarcomagenesis (D). Another means by which severe developmental phenotypes may be eschewed or potential cells of origin tested is by the use of conditional oncogene activation or conditional tumor suppressor ablation, specified either temporally or, by tissue, spatially (E); combinations of tumor suppressor deletions, possible in conditional ablation methods, have been very efficient at driving osteosarcomagenesis.

Bombarding the genome with a chemical, radiation, or an integrating virus will necessarily have pleiotropic effects. There have been other attempts to distress the genome in a more orderly fashion, by genetic manipulation of mice (Figure 2). For instance, aneuploidy can be readily induced via hypomorphic or ablated alleles of certain cell cycle checkpoint and mitotic spindle assembly proteins. Such aneuploidy induced from *UbcH10* disruption [32], *Bub3* and/or *Rae1* disruption [33], or *RanBP2* disruption [34] generates no statistically significant increase in the number of sarcomas arising. In contrast, homozygosity for hypomorphic *Bub1* does increase sarcoma incidence to nearly 15 percent (from a 5-percent background) over the 2-year lifespan typical of these mice [35]. The authors did not identify how many, if any, of these sarcomas were osteosarcomas. In another study the same group added *Bub1*-induced aneuploidy to a background of heterozygosity for a panel of tumor suppressors and identified induced loss of heterozygosity of these tumor suppressors as one mechanism of action for *Bub1* oncogenesis [36].

Insertional mutagenesis can also be driven by transposons in a controlled genetic fashion in murine somatic cells. Similar to oncogenic mutations from insertional retroviruses, most of the tumors that form in *Sleeping Beauty* transposon mice, when it is activated throughout the body, are leukemias and lymphomas [37]. Soft-tissue and a few bone sarcomas have been generated but only with cooperative mutations in *p19Arf* [38].

## 3. Oncogenes

The other mouse models of osteosarcoma that predated the age of gene targeting were derived from insertional viruses that expressed transforming oncogenes. FBJ murine osteosarcoma virus and RFB osteoma viruses were identified from spontaneous murine tumors and the FBR osteosarcoma virus from noncellular extracts from a radiation-induced murine osteosarcoma [39]. All of these viruses were eventually noted to cause high expression of c-*fos*, which became almost the definitional osteosarcoma oncogene [40, 41]. When transgenic mouse technology was possible in the late 1980s, mice transgenic for high c-*fos* expression similarly formed osteosarcomas consistently [42]. Then, c-*Jun*, another powerful oncogene, was found to potentiate c-*fos*-driven osteosarcomagenesis when coexpressed [43]. In screening a number of spontaneous and radiation-induced murine osteosarcomas, a variety of oncogenes were found to be expressed, including c-*abl*, c-*bas*, c-*fos*, K-*ras*, and c-*myc*. No particular oncogene appeared to predominate [44]. So important were these oncogenes felt to be initially that a theory became prominent in the literature during the 1980s and 1990s that radiation-induced osteosarcomagenesis may have as much to do with unsilencing of oncogenes as it does with DNA and chromosomal damage [45, 46].

Perhaps involved with steps other than initiation, other nonviral oncogenes have been found to have potential importance in mouse osteosarcomagenesis. Comparative genomic hybridization on osteosarcomas arising in *p53* heterozygous mice identified a recurrent amplification of mouse chromosomal region 9A1 [47]. This corresponds to an amplification of chromosome 11 common in human osteosarcomas. The antiapoptotic genes *Birc1* and *Birc2*, as well as *matrix metalloproteinase 13* were all found to be upregulated by this amplification. Knockdown of the expression of each impeded tumor growth following engraftments into immunocompromised mice. Further, *ezrin* has been highlighted as a major contributor to the metastatic phenotype in a particular *in vivo*-passaged murine osteosarcoma cell line [48].

No discussion of oncogenes and their role would be complete without mention of the Simian vacuolating virus 40 (SV40), a polyoma virus that readily causes osteosarcoma in hamsters. Expression cassettes from the virus have proven effective in producing osteosarcomas in mice as well [49–52]. As the oncogenes produced function more as a means of inactivating specific tumor suppressors, we will discuss them in greater detail below, acknowledging that, while SV40 T antigens function as tumor suppressors, they are themselves overexpressed and pro-oncogenic, similar to other oncogenes.

## 4. Tumor Suppressor Silencing

### 4.1. Retinoblastoma and p53 Pathways

The first clues that murine osteosarcomagenesis can be driven by *Rb1* and *p53* pathway disruptions came in the high incidence of osteosarcomas in mice with transgenic expression of the SV40 large T antigen [49–52], which is known to bind and silence members of the *Rb1* and *p53* cell cycle checkpoint pathways, effectively disrupting them. Conditional genetic ablation of *Rb1* and *p53* specifically using the *Cre*-lox system has also recently proven sufficient to drive short-latency osteosarcomagenesis [53, 54]. Both research teams, who published essentially identical mouse models based on this technique, concluded that *p53* silencing is necessary and *Rb1* silencing cooperative—but insufficient alone—to drive osteosarcomagenesis.

Germline heterozygosity for *p53* ablation engenders a variety of cancers, but approximately 25 percent of mice heterozygous for *p53* knockout will develop osteosarcomas [55]. While the variety of tumors common in patients with Li-Fraumeni syndrome is generally replicated in heterozygous *p53*-deficient mice, the relative rates of each are quite different. Lymphomas are much more common in murine than in human Li Fraumeni, and carcinomas much less common [56]. Homozygous knockout of *p53* has a lower incidence of osteosarcoma (4 percent), most likely due to the high incidence of early mortality from lymphoma formation [55]. Further studies have reverse-translated specific disease-causing missense mutations from human cancer patients into the mouse and have shown that some can behave more like dominant negative than silent alleles. Heterozygosity for a particular such missense mutation, replacing the arginine at amino acid residue 172 with a histidine, generated a 50% incidence of osteosarcoma with shorter latency than typical for heterozygous *p53* ablation [57]. Other experiments have conditionally ablated *p53* alone in osteoprogenitor cells. Homozygosity for this tissue-specific *p53* silencing efficiently produces osteosarcomas with near-complete penetrance [58].

Germline homozygous deletion of *Rb1* is not compatible with life. Heterozygous Germline *Rb1* disruption does not generate osteosarcoma with any detectably increased incidence, in stark contrast to humans heterozygous for germline *RB* ablation, who have a 500-fold increased incidence of osteosarcoma [59]. *Rb1* heterozygous mice also do not develop retinoblastomas, whereas most humans with the homologous genotype will have bilateral retinoblastomas either congenitally or during infancy. This is possibly due to the reduced stochastic likelihood of losing heterozygosity in retinal cells given that they have undergone fewer cell cycles prior to terminal differentiation in a mouse than in a human. However, mouse chimeras bearing cells with *Rb1* homozygous deletion also do not form retinoblastomas or osteosarcomas. The explanation for this lack of tumorigenesis came in the discovery of a redundancy in the mouse (but not in humans) among the pocket proteins, *Rb1*, *p107*, and *p130* [60]. It was first discovered, with further chimera experiments, that *p107* ablation with *Rb1* ablation efficiently produces retinoblastomas and early mortality in mice [61]. Similar chimera experiments with homozygous *p107* deletion and heterozygous *Rb1* deletion were performed to permit appreciation of the broader tumor spectrum, typical of humans heterozygous for *Rb1* disruption. Eight of the 53 chimeric mice observed developed osteosarcomas, most of which had lost heterozygosity for *Rb1* in the tumor cells [62]. These data, although overlooked in recent articles arguing from mouse data for the predominance of the *p53* pathway as critical and *Rb1* as only cooperative, suggest that a slight variation in pocket protein biology between mouse and human may have masked the importance of the retinoblastoma pathway overall in mouse osteosarcomagenesis.

The *INK4a/ARF* locus in the mouse and human has implications for both the *p53* and *Rb1* pathways. Although disruption of at least the *INK4a* portion is common in human osteosarcomas that otherwise lack *RB* silencing [63], it has received little attention in the mouse. Beginning from cultured murine mesenchymal progenitors, *INK4a/ARF* disruption in tandem with c-*myc* oncogene expression has proven sufficient to generate cells that will form osteosarcomas when injected orthotopically into syngeneic mice [64]. *In vivo* osteosarcomagenesis experiments have yet to be performed with respect to *INK4a/ARF*.

### 4.2. Osteoprogenitor Differentiation Program Factors

Speculation has reigned over the consideration that *Runx2*, the definitional preosteoblast transcription factor, functions as a tumor suppressor gene in osteosarcoma. Other *Runx* family genes have been implicated in cancers [65]. The semidifferentiated state of osteoid producing osteosarcoma cells certainly raises questions about the *Runx2*-mediated juncture in osteoblast differentiation. While Runx2-driven osteoblast differentiation is clearly truncated in osteosarcoma, expression tends to be higher than in osteoblasts and correlates with a poor prognosis [66, 67]. Experiments in mice and using *ex vivo* murine cells in culture have identified interactions between *Runx2* and both *p53* and *Rb1* defining the early stages of the osteoblast differentiation program [58, 68]. One of the ways that *Rb1* loss is felt to prime for osteosarcomagenesis is by disrupting the feed-forward cycle between *Runx2* and *p*27^*kip*1^ [69]. Disruption of this feed-forward loop results in osteoprogenitors failing to terminally differentiate and exit cell cycle [70]. This explains the dysfunctional presence of *Runx2* in human osteosarcomas.

*Prkar1*α** was recently proposed as another tumor suppressor gene involved in osteosarcomagenesis in the mouse [71]. Disruption of this gene reduced the latency to osteosarcomagenesis in mice transgenic for SV40 large T and small t antigens expressed by the osteocalcin promoter. *Prkar1*α** is involved with osteoblast-osteoclast *RANK/RANKL* interactions and osteoclast differentiation and activation programs. A potential challenge to the role of *Prkar1*α** disruption more generally in osteosarcomagenesis is that the differentiated osteoblasts in which this model initiate T-antigen-mediated genomic distress are also more likely to be embedded within tight anatomic constraints, perhaps overemphasizing their need to recruit bone destroying cells to permit geographical expansion and invasiveness. *Prkar1*α** may be a critical tumor suppressor for this particular model and perhaps a subset of osteosarcomas, but not critical overall. A correlation between human osteosarcoma expression of *Prkar1*α** and response to chemotherapy was also discussed in the same paper but rendered somewhat equivocal results [71].

### 4.3. Other General Tumor Suppressors

 One recent finding is that *Wnt-inhibitory factor-1* (*Wif1*) functions as a tumor suppressor in osteosarcomas. It was identified among genes frequently silenced by promoter hypermethylation in a panel of human osteosarcoma samples [72]. Mice homozygous for targeted deletion of *Wif1* were noted to have a slight tendency toward osteosarcomagenesis (2 of 13 mice); when exposed to beta-emitting calcium 45 radioisotope, the typical latency to osteosarcomagenesis was shortened by two months compared to wild-type controls [72]. *Wif1* knockout essentially enhances *Wnt* signaling, offering the possibility of a therapeutic target in *Wnt* signaling itself. 

*Hypermethylated in cancer 1* (*Hic1*), by its name, is another epigenetically modified locus that has also been reported to have a tumor suppressor function in osteosarcoma. Mice homozygous for both *p53* and *Hic1* deletion have a similar life expectancy overall, compared to *p53* deletion alone, but much more frequently develop osteosarcomas [73]. *Hic1* and *p53* are located in close proximity on mouse chromosome 11 and human chromosome 17. Heterozygous deletion of *trans* alleles of *p53* and *Hic1* results in increased incidence of metastatic osteosarcomas over either allele alone [73]. Interestingly, in human tumors, *Hic1* is only hypermethylated in tumors with *p53* mutations, suggesting that it is a dependent, necessarily secondary tumor suppressor.

*Wwox* is a tumor suppressor gene located in one of the most fragile loci in the mammalian genome. While heterozygotes can be induced to generate a variety of tumors, mice homozygous for Germline deletion of *Wwox* form bone surface lesions suggestive of chondroblastic osteosarcomas and then die at a very young age with no additional tumorigenesis noted [74]. *Wwox* expression has been assessed in human tumors as well, where it appeared to have reduced or absent expression in a majority of osteosarcomas and increased in response to chemotherapy [75].

Mice bearing deletion of the *Xpa* gene and heterozygosity for *p53* developed more frequent osteosarcomas than controls when exposed to diethylstilbestrol, but *Xpa* knockout alone was not different than controls, suggesting that *p53* played a more critical role in these experiments [76]. Although *Xpa* disruption has only received minimal attention in human osteosarcomas, one investigation of polymorphisms in it and other nucleotide excision repair genes found shorter event-free survival from osteosarcoma in cisplatin-treated patients bearing a polymorphism in *ERCC2*. Similarly, cooperative disruption of *p53* and *Brca2* generated osteosarcomas in mice, but the increased incidence over baseline was primarily attributed to the former tumor suppressor [77]. A single case of osteosarcoma in a patient with a germline *Brca2* mutation has been reported, but the association has not been investigated further [78].

### 4.4. Surprises

After congenital bilateral retinoblastoma and Li Fraumeni syndromes (from *RB* and *p53* heterozygous inactivation in the germline, resp.), the next highest incidence of osteosarcoma in humans occurs in a heritable syndrome called Rothmund-Thomson syndrome, which results from homozygosity for *RECQL4 helicase* ablation. A model of *Recql4 helicase* disruption has been generated in the mouse by gene targeting, but osteosarcomas have not been identified, despite recapitulation of other features of the autosomal recessive heritable disorder [79, 80]. Because *RECQL4 helicase* has not been found to be mutated or deleted in sporadic human osteosarcomas, it is felt that its disruption represents more of a route to generate genomic instability generally than the silencing of a specific tumor suppressor important to osteosarcomagenesis.

One of the first gene-targeted mouse models noted to develop frequent osteosarcomas is the *merlin* or *Nf2* knockout [81]. There is no human correlate to this. *NF2* deletion has not been detected in human osteosarcomas [82]. Surprisingly, many attempts at acoustic schwannoma (the tumor that predictably arises in *NF2* heterozygous humans) formation in these mice have been thwarted by their strong propensity toward osteosarcomagenesis. The only direct link that has fit with other knowledge of osteosarcoma so far is that the *Nf2* and *p53* genes are in relative proximity in the genome and the osteosarcomas that form often lose one or both alleles of *p53* as well. However, overexpression of *ezrin* is important to human osteosarcomagenesis, especially development of the metastatic phenotype [48]. *Nf2*, or *merlin*, is named as the moesin_ezrin_radixin_like protein. A possible relationship between *merlin* disruption in the mouse and *ezrin* overexpression in humans has yet to be deciphered for osteosarcomagenesis.

## 5. Tissue of Origin

The anatomic placement of most successfully osteosarcomagenic chemicals in the metabolically active metaphyses of long bones and the natural homing of alpha-emitting heavy metals to the same both make some philosophical comment on the osteosarcoma tissue of origin. That said, a wide variety of cell lineages are typically active even within this constrained anatomy. Osteosarcomas are pathologically defined as malignant appearing cells producing bone, but this can be a small minority of the overall tissue volume of a given tumor. There can be osteoblastic, chondroblastic, fibroblastic, and telangiectatic tissue types in any single osteosarcoma. This fact has long raised the question of what cell within the bone is the cell in which the program of oncogenesis begins: marrow stromal cells, mesenchymal progenitor cells, stem cells, differentiated osteoblasts, or hematopoietic progenitors.

**Figure 3:**
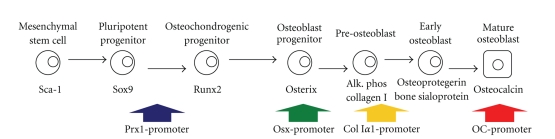
Osteosarcomagenesis has been tested from a range of cells of origin along the differentiation pathway of osteoblasts. *Cre*-recombinase expression driven from the *Prx1*, *osterix*, and *collagen 1a1* promoters has generated osteosarcomas in mice when used for the conditional inactivation of *p53* with or without *Rb1*. The osteocalcin promoter, defining a late stage of osteoblast differentiation usually not expressed in osteosarcoma cells directly, has proven sufficient for osteosarcomagenesis, when used to drive SV40 large and small T antigens.

The tools for conditional gene disruption and oncogene activation have greatly enhanced our capacity to test for specific cell types of origin (Figure 3). Of course, all that can really be tested with any of these experiments is osteosarcomagenic sufficiency or lack thereof of the induced derangement in the given cell expressing a specific promoter. A variety of promoters have proven sufficient to drive osteosarcomagenesis enacting the specific conditional derangements planned. SV40 transgenic promoters included the alpha-amylase promoter [49, 52], the heat shock protein 70 promoter from Drosophila [50], and the myelin basic protein promoter [51], each of which managed to generate osteosarcomas in mice, confirming that each was expressed in at least some sufficient cells of origin.

With *Cre*-lox conditional ablation of *p53* as the given derangement, different mesenchymal and preosteoblast promoters have proven sufficient for osteosarcomagenesis. The first tried was a *collagen 1*α*1* promoter fragment, which also generated a number of lymphomas, suggesting leakiness into earlier mesenchyme as the possible source of sufficient originating cells [58]. Others have used the *Osterix* promoter to drive *Cre*-mediated conditional ablation of *p53* with or without tandem *Rb1* conditional ablation [53, 54]. This should be expressed in committed but not yet terminally differentiated osteoprogenitors. These mice formed some adipocytic tumors in addition to the high incidence of osteosarcoma, also suggesting some contribution of either dedifferentiation after tumor suppressor silencing or promoter leakiness into earlier multipotent mesenchyme. Finally, *Prx1*-*Cre* was used to drive tumor suppressor silencing in lateral plate mesoderm cells and what certainly remains pluripotent mesenchymal progenitors [83]. These mice developed a number of soft-tissue sarcomas as well as osteosarcomas, highlighting the originating cells' pluripotency.

The genetic mouse model initiating osteosarcomagenesis in the most differentiated cells uses the *osteocalcin* promoter to express large and small T antigens from SV40 [71]. Clearly, a sufficiently strong sledge hammer to the genome can produce osteosarcomas even from what should be cells that have exited cell cycle. It may be that a lesser hit in the same cells would prove insufficient to drive osteosarcomagenesis. All we know is that we have not pushed against the utter extreme yet for osteosarcomagenesis from more differentiated cells. Further, we are no closer to knowing which of these sufficient osteoprogenitors is most frequently the actual cell of origin in humans.

## 6. Host Factors

Although this has received the least attention so far from investigators in the field, mice also have been manipulated to identify the effects of the background strain or presence of specific germline expressions or disruptions on other methods of inducing osteosarcomagenesis. Not long after the report of the first targeted disruption of *p53* in the mouse, a paper compared the cancer spectrum and incidence between the 129/Sv strain and a mixed C57BL/6+129/Sv strain, finding no significant difference for osteosarcoma specifically [84]. The rates of plutonium-induced osteosarcomagenesis were not significantly different between C3H/He, C57BL/6, and B6C3F_1_ strains in another study [85]. 

 Surgical hypophysectomy in mice prior to implantation of grafted tumors suggested the role of *Igf1* in osteosarcomagenesis [86]. Later host mice engineered genetically to have *Igf1* gene ablation demonstrated similar results [87, 88]. Certainly, these were models intended to ask specific questions about pathway contributions rather than identify the typical etiologies behind human osteosarcoma, but they highlight an area of research that is already taking off with respect to other cancers and host-tumor interactions. With the technology for deep sequencing of polymorphisms and their variance between strains of mice, we may eventually be able to understand much about the more subtle host genetic contributions to risk of osteosarcoma.

One notable and unusual study of host factors in osteosarcomagenesis came in the toxicity screening of teriparatide or PTH(1-34). Fischer 344 rats developed high rates of osteosarcoma from prolonged administration of the parathyroid hormone biological analog, even at lower doses [89]. No human correlate to this has been encountered, with only two osteosarcoma cases identified out of over 430,000 human individuals treated with the drug [90]. Treatment of human and murine cells as well as mice *in vivo* with teriparatide-generated DNA double-stranded breaks and chromosomal abnormalities [91]. While this may contribute mechanistically to osteosarcomagenesis in the rat, the mechanism by which PTH(1-34) generates DNA and chromosomal damage is not yet elucidated. Interestingly, treatment of Fischer 344 rats with the full-length PTH(1-84), which includes the C-terminal domain, did not induce osteosarcomas as efficiently at lower doses [92].

## 7. Conclusions

With the persistent elusiveness of a precursor lesion for osteosarcoma, even in very predictable genetic mouse models of the disease, we continue to learn most of what we know by candidate gene or candidate insult approaches, testing always for sufficiency in osteosarcomagenesis, but never necessity. We know that appropriately located chemical carcinogens or applied radiation rays or alpha-emitting radioisotopes have all proven sufficient to drive osteosarcomagenesis by driving mutagenesis and chromosomal instability, but genetically induced aneuploidy alone is not usually sufficient. We have learned that, when appropriately accounting for a mouse-specific *Rb1* redundancy from *p107*, disruption of either the *Rb1* pathway or *p53* alone in the mouse generally is sufficient to drive osteosarcomagenesis. Their combination is alarmingly efficient, consistent with the observation from human osteosarcomas that both pathways are usually disrupted by some means in tumors. We have learned that these disruptions can initiate osteosarcomagenesis in undifferentiated mesenchymal progenitors or even committed cell types.

Other pathways, oncogenes, and tumor suppressors have been identified, some of which are clearly dependent on other disruptions or the application of other genetic insults to drive osteosarcomagenesis. No doubt, improved oncogenomic techniques will inspire yet new mouse models and the testing of the sufficiency of newly identified tumor suppressor disruptions and oncogene activations in the mouse. The new fields of host and niche biology as the environments in which cancers initiate and develop can also be expected to bring new knowledge to osteosarcomagenesis as well as further innovative experimentation in the mouse. 

There remain challenges to the use of the mouse as a model organism for human osteosarcomagenesis. First, rodents more readily form osteosarcomas than do humans. Second, there are a number of specific pathways that are difficult to translate between the two. Other challenges, such as the lack of true lamellar bone in the mouse, highlight critical discrepancies in healthy bone biology between the species. Many scientists argue that genetically engineered or transplanted syngeneic osteosarcomas in rats, which have lamellar bone structure, or spontaneous osteosarcomas in canines may provide better preclinical models for drug testing and so forth. Nonetheless, the mouse as preclinical model may be less enlightening than the mouse as testing platform for the induction of osteosarcomas. The breadth and depth of genomic understanding of the mouse and facility with which it can be experimentally manipulated will not soon be replicated in any other mammalian species. While care must be taken in interpreting results in the mouse, attending to interspecies variations, that same attention can broaden our understanding of this complex disease even further. For example, specific biological eccentricities such as the pocket protein redundancy in mice have not prevented illumination of the human disease; they just require more careful attention to all the available literature. In similar fashion, perhaps further interrogation of *Nf2* in murine osteosarcomagenesis may yet highlight pathways that are important, but unrecognized in the human diseasem.

Although mice and humans are decidedly different in many ways, most of the knowledge gained from mouse modeling has been validated in human clinical samples and cell lines. Clearly, the opportunity to prospectively test hypotheses in mouse-modeled osteosarcomagenesis is unique. There is doubtless much that remains to be learned from the mouse with regard to osteosarcoma initiation, progression, and metastasis.
